# 支链聚乙烯亚胺辅助硼酸功能化磁性纳米粒子的制备及其用于人参皂苷的特异性富集

**DOI:** 10.3724/SP.J.1123.2020.11005

**Published:** 2021-06-08

**Authors:** Xue LI, Zhifeng YAN, Longzhu LI, Tao MA, Yang CHEN

**Affiliations:** 1.蚌埠医学院药学院, 安徽 蚌埠 233030; 1. School of Pharmacy, Bengbu Medical College, Bengbu 233030, China; 2.蚌埠医学院公共基础学院, 安徽 蚌埠 233030; 2. School of Public Basic Courses, Bengbu Medical College, Bengbu 233030, China

**Keywords:** 聚乙烯亚胺, 硼亲和, 磁性纳米粒子, 高效液相色谱, 人参皂苷, polyethyleneimine, boronate affinity, magnetic nanoparticles, high performance liquid chromatography (HPLC), ginsenosides

## Abstract

为了实现更高效的人参皂苷富集,以硼亲和色谱为核心,结合支链聚乙烯亚胺放大硼酸配基数量,合成了支链聚乙烯亚胺辅助硼酸功能化磁性纳米粒(PEI-BA-MNPs),用于实际样品中人参皂苷的选择性富集,结合高效液相色谱,建立了一种分析实际样品中的人参皂苷的方法。以人参皂苷Re为代表,在优化的磁性固相萃取的条件下,该方法在50~800 μg/L的范围内呈现良好的线性,线性相关系数(*R*^2^)为0.9681。添加水平在0.1~10 mg/L时,回收率为91.5%~117.3%,相对标准偏差为7.2%~13.4%。由于所得材料对于人参皂苷的高亲和力,经所建立的方法富集过后,人参皂苷Re的灵敏度提高了约50倍。同时,所得材料重复使用5次以后还可以保持至少72%的原始吸附量。最后,将该方法用于启脾口服液中人参皂苷Re的含量分析,并与2015版《中国药典》的标准方法做对比。结果显示,所建立的方法检测出的人参皂苷Re含量为0.27%,虽然与标准方法测得的含量(0.31%)有些微差距,但该法极大地节约了实际操作中样品前处理的步骤和时间。结果表明,所制得的PEI-BA-MNPs可以用作磁性固相萃取吸附剂实现实际样品中人参皂苷的选择性富集。该方法亲和力强,选择性好,灵敏度高,操作快速简便且准确度高,具有很大的应用价值和发展前景。

## Abstract (en)

Panax ginseng has a 5000-year-long history as a traditional herbal medicine in Eastern Asia and North America. It is also known as crown jewel in traditional Chinese herbs because of its wide pharmacological properties. Ginsenosides, a class of saponins containing triterpene aglycones and various sugar moieties, are the main active components of ginseng. Considering the low abundance of ginsenosides and other abundant interferences, separation of ginsenosides is essential prior to further analysis. Recently, our group demonstrated the potential of a boronate affinity material for the selective enrichment of ginsenosides. However, conventional boronate affinity materials suffer from an apparent drawback. The binding strength of boronic acids toward *cis*-diol-containing compounds is low, with dissociation constants (*K*_d_) ranging from 10^-1^ to 10^-3^mol/L. Thus, it is necessary to develop boronate affinity materials with high binding strength. In this study, we developed polyethyleneimine (PEI)-functionalized boronate affinity magnetic nanoparticles (BA-MNPs) for the selective enrichment of ginsenosides. Branched PEI was applied as a scaffold to amplify the number of boronic acid moieties, while 3-formylphenylboronic acid, which shows high affinity toward *cis*-diol-containing molecules, was used as the affinity ligand. In addition, the presence of the multi-glycan structure of ginsenoside leads to higher binding affinity between the PEI-BA-MNPs due to the synergistic multivalent binding effect. Combining with high performance liquid chromatography, a method for the selective analysis of ginsenosides was established. With ginsenoside Re as the representative and under the optimized conditions for magnetic solid-phase extraction, the developed method showed good linearity in the range of 50-800 μg/L, with a linear correlation coefficient (*R*^2^) of 0.9681. At different spiked levels (0.1-10 mg/L), the recoveries were in the range of 91.5%-117.3%, and the relative standard deviations (RSDs) ranged from 7.2% to 13.4%. Since the PEI-BA-MNPs exhibited significantly improved binding strength toward ginsenosides, they could extract trace glycoproteins. After enrichment, a 50-fold improvement in the sensitivity was achieved. In addition, the PEI-BA-MNPs maintained at least 72% of their original binding capacity after five consecutive uses. Finally, the developed method was applied to the determination of ginsenoside Re in commercial medicine (Qipi oral liquid). As opposed to the tedious and time-consuming sample preparation in the standard method (Pharmacopoeia of the People’s Republic of China, 2015; ChP2015), the present protocol allowed for direct enrichment of the diluted commercial medicine with PEI-BA-MNPs. The magnetic separation made the overall experiment much simpler than the standard ChP2015 method. After washing and elution, the enriched ginsenoside Re was eluted and subjected to HPLC-UV analysis. The results obtained with the developed method (0.27%) were similar to those of ChP2015 (0.31%). We have experimentally demonstrated that PEI-BA-MNPs are ideal affinity sorbents for the selective enrichment of ginsenosides owing to their significant advantages, including high affinity, excellent selectivity, easy manipulation, high binding capacity, and fast binding equilibrium. As many saponins contain sugar side chains, we foresee a promising prospect for the proposed method in real-world applications.

人参为五加科植物人参(Panax ginseng C. A. Mey.)的干燥根和根茎,是我国传统的名贵中药材,最早出现在《神农本草经》中,具有大补元气、固脱、生津、安神和益智等功效^[1]^。人参的化学成分较为复杂,研究已表明,人参皂苷为人参中的主要活性成分^[2]^,具有抗肿瘤^[3,4,5,6]^、调节心脑血管系统^[7]^、调节中枢神经系统^[8,9]^、调节免疫系统^[10]^、抗疲劳^[11]^、抗氧化^[12]^、抗辐射^[13]^、抗衰老^[14]^和抗糖尿病^[15]^等多种药理作用,但其在人参中含量较低,测定时易受到其他组分的干扰。因此,采用合适的人参皂苷富集方法对人参皂苷分析具有十分重要的意义。

硼亲和材料是一种选择性分离富集顺式二羟基生物分子的功能性材料^[16]^,硼酸配体可以可逆地与含顺式二羟基的化合物结合,如糖蛋白^[17]^、聚糖、糖类、核苷和核苷酸。这些含顺式二羟基的生物分子在蛋白质组学、代谢组学、糖组学、糖生物学等当前科学前沿中是重要的目标分子^[18]^。但单一硼酸对含有顺式二羟基化合物的结合强度相对较弱,因此,用传统的硼酸盐亲和材料无法胜任低浓度的顺式二羟基分子的选择性富集任务^[19]^。支链聚乙烯亚胺(PEI)可以作为一种较好的载体来扩增材料表面可供修饰的官能团,使硼酸位点大量增加^[20]^。人参皂苷分子多数都具有多糖链结构,这也为多位点协同结合提供了基础(具体结合原理见图1)。Li等^[21]^制备了PEI修饰硼亲和毛细管整体柱,结果表明其对于糖蛋白的结合力较单一硼酸提高了1~2个数量级。同时,纳米颗粒在分离、催化、传感器和药物传递等领域引起了广泛的关注。其中磁性Fe_3_O_4_纳米颗粒由于其良好的生物相容性、超顺磁性、低毒性和易于操作等特性在样品制备中得到广泛关注^[22,23,24,25]^。最近,本课题组制备了硼酸修饰的介孔硅纳米颗粒,并成功用于人参皂苷的选择性富集^[26]^。但由于单一硼酸与人参皂苷分子较低的结合力,此方法尚不能满足中成药中人参皂苷的分析需求,因此需要选用与人参皂苷分子具有更高亲和力的功能化材料。

**图 1:**
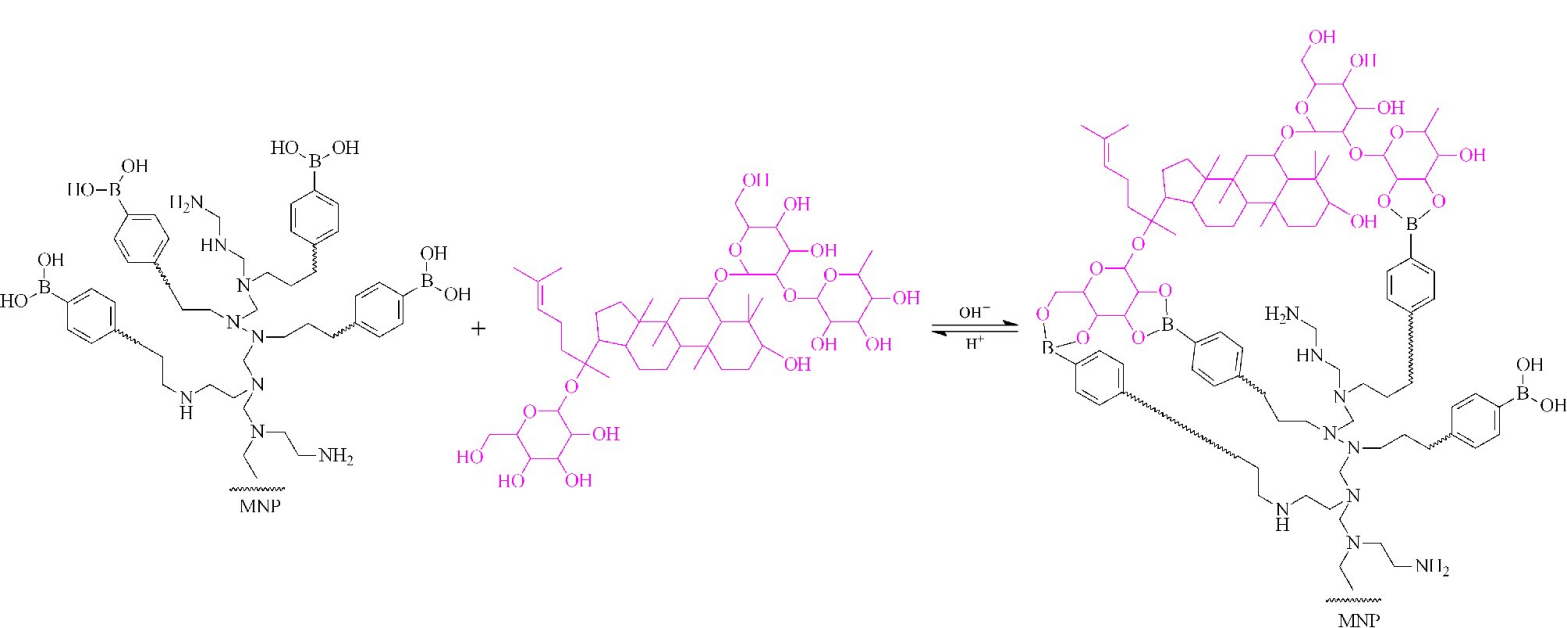
支链聚乙烯亚胺修饰的硼亲和磁性纳米粒子(PEI-BA-MNPs)与人参皂苷Re结合示意图

本研究中,以Fe_3_O_4_纳米颗粒为磁核,采用支链聚乙烯亚胺作为支架放大后修饰的硼酸(3-甲酰基苯硼酸)官能团数目,合成了一种支链聚乙烯亚胺修饰的硼亲和磁性纳米粒子(PEI-BA-MNPs),采用磁性固相萃取的方法实现了人参皂苷的高效富集。以人参皂苷Re为例,对于材料性能进行验证,同时结合高效液相色谱定量分析,测定实际样品启脾口服液中的人参皂苷含量。

## 1 实验部分

### 1.1 仪器、试剂与材料

透射电子显微镜(TEM)JEM1010系统(日本东京JEOL);超微量紫外可见分光光度计(美国Thermo Fisher公司); HPLC系统为Waters公司,该系统由1525二元溶剂泵、2998 PDA检测器和2707自动进样器组成。

PEI(相对分子质量10000)购自阿法埃莎(中国)化学有限公司,六水合三氯化铁、乙二醇、无水乙酸钠、1,6-己二胺、5%(v/v)戊二醛甲醇溶液、无水甲醇、乙腈、磷酸、正丁醇、三氯甲烷、氰基硼氢化钠、3-甲酰基苯硼酸、乙酸、磷酸钠(十二水)、氨试液、腺苷、脱氧腺苷、葡萄糖、果糖均为分析纯,购自天津市大茂化学试剂厂。实验用水均为Milli-Q纯水系统制备的超纯水。人参皂苷Re、人参皂苷Rb1、原人参三醇、原人参二醇购自上海源叶生物科技有限公司,均为分析标准品,纯度≥98%。

启脾口服液(吉林益民堂制药有限公司)购自当地药店(蚌埠)。

### 1.2 磁性纳米粒子的制备

1.2.1 Fe_3_O_4_磁性纳米粒子的制备

首先将FeCl_3_·6H_2_O(2.0 g)溶解在60 mL的乙二醇中,形成澄清的橙黄色溶液。然后,向上述溶液中添加4.0 g无水乙酸钠和13.0 g 1,6-己二胺。将混合物剧烈搅拌30 min,并密封在100 mL反应釜中,在198 ℃下反应6 h,得到氨基修饰的Fe_3_O_4_磁性纳米粒子(AMNPs)。

1.2.2 支链聚乙烯亚胺修饰的磁性纳米粒子(PEI -MNPs)的制备

将200 mg AMNPs加入40 mL 5%(v/v)戊二醛甲醇溶液中,在室温下机械搅拌12 h。所得戊二醛活化的MNPs用无水甲醇洗涤4次后,先加入20 mL无水甲醇,然后用超声波分散在含有0.5 g PEI的20 mL无水甲醇中。在室温下将混合物机械搅拌12 h。随后每6 h加入10 mL氰基硼氢化钠甲醇溶液(20 g/L),持续反应12 h。用磁铁收集反应得到的PEI-MNPs,洗涤后进行真空干燥。将获得的PEI-MNPs储存以备进一步使用。

1.2.3 PEI-BA-MNPs和硼亲和磁性纳米粒子(BA-MNPs)的制备

首先将200 mg PEI-MNPs用超声分散在30 mL无水甲醇中,随后加入10 mL 3-甲酰基苯硼酸无水甲醇溶液(50 g/L)中,再加入10 mL氰基硼氢化钠甲醇溶液(30 g/L),搅拌24 h。用磁铁收集反应得到的PEI-BA-MNPs,洗涤3次后进行真空干燥。将获得的PEI-BA-MNPs储存以备进一步使用。对于BA-MNPs的合成,使用AMNPs代替PEI-MNPs,其余步骤保持不变。

### 1.3 磁性固相萃取过程

称取3 mg的PEI-BA-MNPs,先加入0.5 mL空白溶剂(含30%甲醇和70% pH 8.5的磷酸盐缓冲液),超声使PEI-BA-MNPs均匀分散,再加入等量的人参皂苷标准溶液,混合均匀后,振荡60 min,弃去上清液,用空白溶剂清洗,加入200 μL的0.1 mol/L乙酸,振荡60 min,吸取上清液,在203 nm处测定紫外吸收度。

### 1.4 亲和力测定

PEI-BA-MNPs对于人参皂苷的亲和力采用Scatchard拟合进行分析。根据以下Scatchard方程计算解离常数(*K*_d_)和表观最大结合容量(*Q*_max_):

*Q*_e_/*C*_s_=(*Q*_max_-*Q*_e_)/*K*_d_

其中*Q*_max_和*K*_d_分别是饱和结合容量和解离常数,*Q*_e_是人参皂苷在平衡时与PEI-BA-MNPs和BA-MNPs的结合量,*C*_s_是吸附平衡时的自由浓度。*K*_d_和*Q*_max_的值可以根据*Q*_e_/*C*_s_与*Q_e_*曲线的斜率和截距来计算。

### 1.5 启脾口服液样品预处理

本工作方法:将启脾口服液用含30%甲醇和70% pH 8.5的磷酸盐缓冲液稀释100倍后,按照1.3节中所述过程进行萃取。

2015版《中国药典》(ChP2015)标准方法:精密量取启脾口服液50 mL,加入三氯甲烷振摇提取3次,每次30 mL,弃去三氯甲烷提取液,水液中加入水饱和的正丁醇振摇提取5次(50、30、30、20、20 mL),合并正丁醇提取液,加氨试液洗涤4次,每次50 mL,弃去氨试液,再加正丁醇饱和的水轻轻振摇洗涤2次,每次50 mL,弃去水洗液,正丁醇液回收溶剂至干,残渣加甲醇溶解并转移至5 mL量瓶中,加甲醇稀释至刻度,摇匀,滤过,取续滤液,即得。

### 1.6 高效液相色谱条件

色谱柱:Inertsil ODS-3色谱柱(150 mm×4.6 mm, 5 μm),检测波长:203 nm,流速:1.3 mL/min。流动相为1%(v/v) H_3_PO_4_(A)和ACN(B),梯度如下:0~30 min, 19%B; 30~35 min, 19%B~24%B; 35~60 min, 24%B~40%B; 60~70 min, 40%B,进样量为20 μL。所有流动相在使用前通过0.45 μm的滤膜过滤,并在超声中脱气。

## 2 结果与讨论

### 2.1 磁性纳米粒子的表征

用TEM对制备的PEI-BA-MNPs进行了形貌表征。如图2a所示,TEM图像显示PEI-BA-MNPs具有良好的分散性和相对均匀的尺寸分布,平均直径约为100 nm。如图2b所示,PEI-BA-MNPs在水中均匀分散,并被外部磁铁迅速吸引到容器壁上。当去除磁体时,聚集的纳米粒子可以通过超声重新分散。首先采用腺苷和脱氧腺苷这一对化合物对PEI-BA-MNPs的硼亲和作用进行表征,按照1.3节中的方法对腺苷和脱氧腺苷标准溶液进行萃取和解吸,所得溶液在260 nm处测定紫外吸收度,结果(见图2c)显示腺苷的紫外吸收度远大于脱氧腺苷,说明所合成的PEI-BA-MNPs对含有顺二羟基分子具有选择性。

**图 2:**
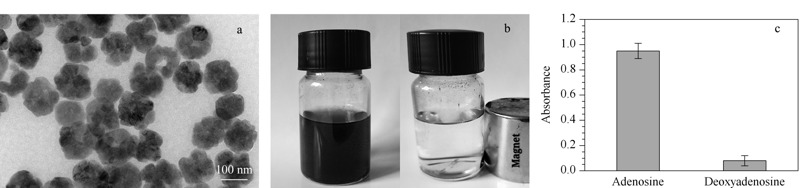
PEI-BA-MNPs的(a)透射电子显微镜照片、(b)磁分离照片和(c)PEI-BA-MNPs结合腺苷、脱氧腺苷的能力考察(*n*=3)

### 2.2 磁性固相萃取条件的优化

2.2.1 吸附时间

吸附时间是固相萃取技术中的一个重要参数,一般情况下,在吸附剂与目标物未达到结合平衡时,吸附剂的结合能力会随着吸附时间的增加而增加,在保证富集效果的前提下,应尽量缩短吸附时间。

按照1.3节中的过程进行萃取,如图3a所示,分别考察了吸附时间为5、10、20、30、60、360 min时磁性纳米粒子的结合能力,结果表明,在5~60 min内,随着吸附时间的增加,磁性纳米粒子的吸附能力逐渐增大;当吸附时间为60 min时,结合能力达到最大;之后随着吸附时间增加,磁性纳米粒子的结合能力变化不大。因此,本工作选择60 min作为最佳吸附时间。

**图 3:**
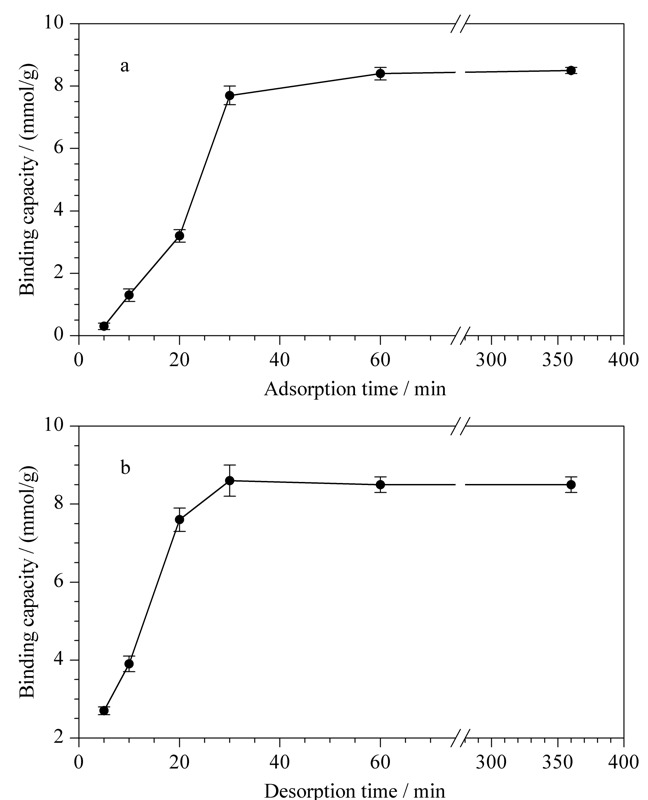
(a)吸附时间和(b)解吸时间对PEI-BA-MNPs结合人参皂苷Re(1 g/L)容量的影响(*n*=3)

2.2.2 解吸时间

如图3b所示,分别考察了解吸时间为5、10、20、30、60、360 min时磁性纳米粒子的结合容量,结果表明,在5~30 min内,随着解吸时间的增加,磁性纳米粒子的结合容量逐渐增大;当解吸时间为30 min时,结合能力达到最大;之后随着解吸时间增加,磁性纳米粒子的结合能力几乎不再变化。因此,基于以上实验结果,本工作选择30 min为最佳解吸时间。

### 2.3 亲和力

以人参皂苷Re为例,对制得的PEI-BA-MNPs和BA-MNPs进行Scatchard分析,如图4a所示,人参皂苷Re在平衡时与PEI-BA-MNPs的结合量*Q*_e_远高于与BA-MNPs的结合量。

**图 4:**
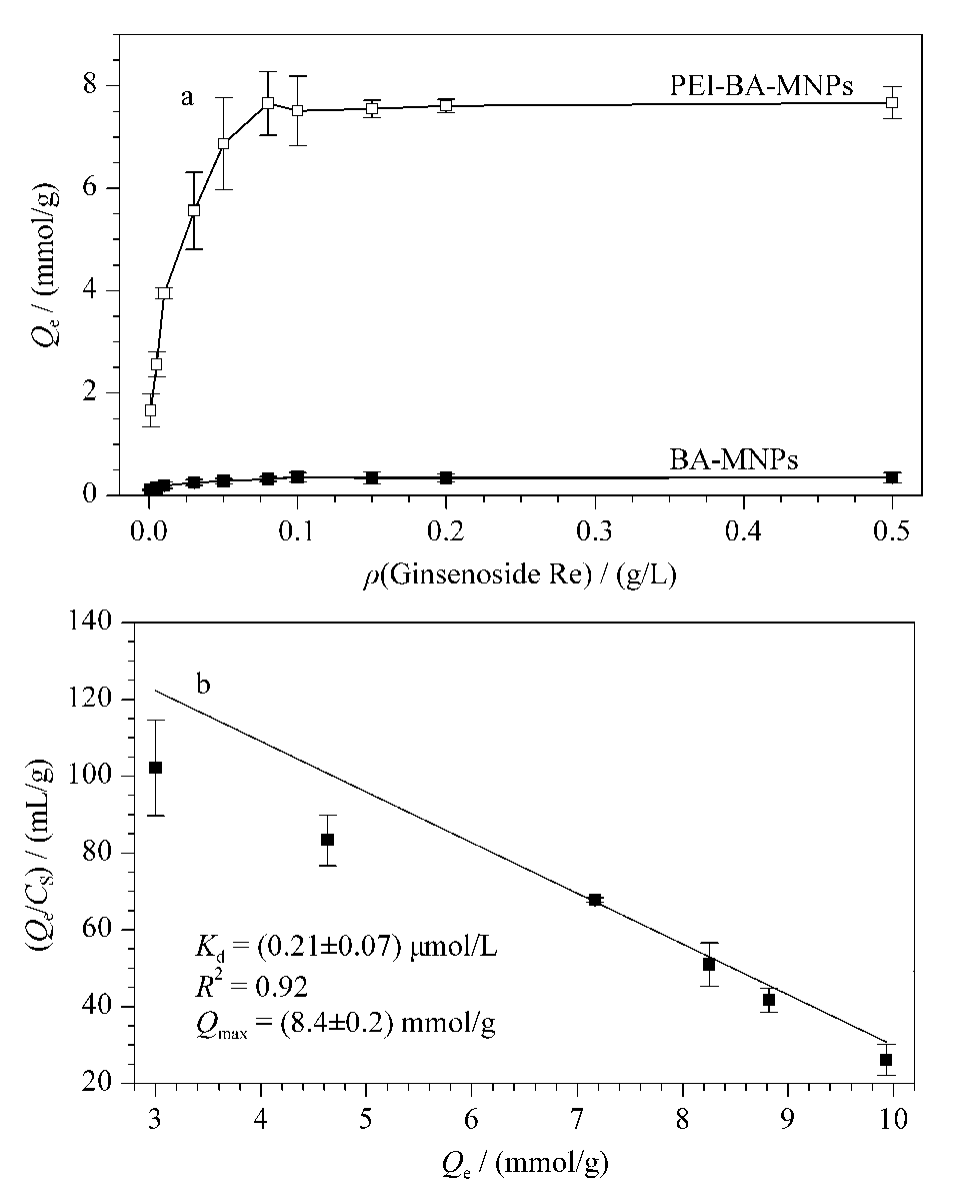
(a) BA-MNPs与PEI-BA-MNPs对人参皂苷Re 的等温吸附线及(b)PEI-BA-MNPs对人参皂苷Re吸附的Scatchard分析(*n*=3)

根据图4b中的Scatchard分析,PEI-BA-MNPs的*K*_d_值为(0.21±0.07) μmol/L; *Q*_max_为(8.4±0.2) mmol/g。和硼酸与单糖的作用力相比^[26,27]^,其对于人参皂苷的亲和力有了明显的增加,这为PEI-BA-MNPs用于人参皂苷高效富集提供了理论依据。

### 2.4 选择性

本工作以人参皂苷Re和Rb1为代表,研究PEI-BA-MNPs的选择性。在PEI-BA-MNPs结合了人参皂苷(Re或Rb1, 0.5 g/L)之后,将PEI-BA-MNPs加入高浓度的竞争物质溶液(空白溶剂、葡萄糖、果糖、原人参三醇、原人参二醇,5 g/L)后振荡1 h,随后测定PEI-BA-MNPs对于人参皂苷(Re或Rb1)的吸附量。如图5所示,PEI-BA-MNPs与Re或Rb1的结合基本不受干扰,将加入空白溶剂的吸附量设为100%,加入其他高浓度竞争物质的吸附量均保持了原始吸附量的95%以上。对于葡萄糖、果糖而言,由于只具有一个顺式二羟基结构,其与材料之间的亲和力显著弱于含多个顺式二羟基结构的人参皂苷。对于原人参三醇、原人参二醇而言,其不具有顺式邻二羟基结构,因而与材料之间无亲和力。此现象为使用PEI-BA-MNPs对实际样品中的人参皂苷进行选择性富集提供了可能。

**图 5:**
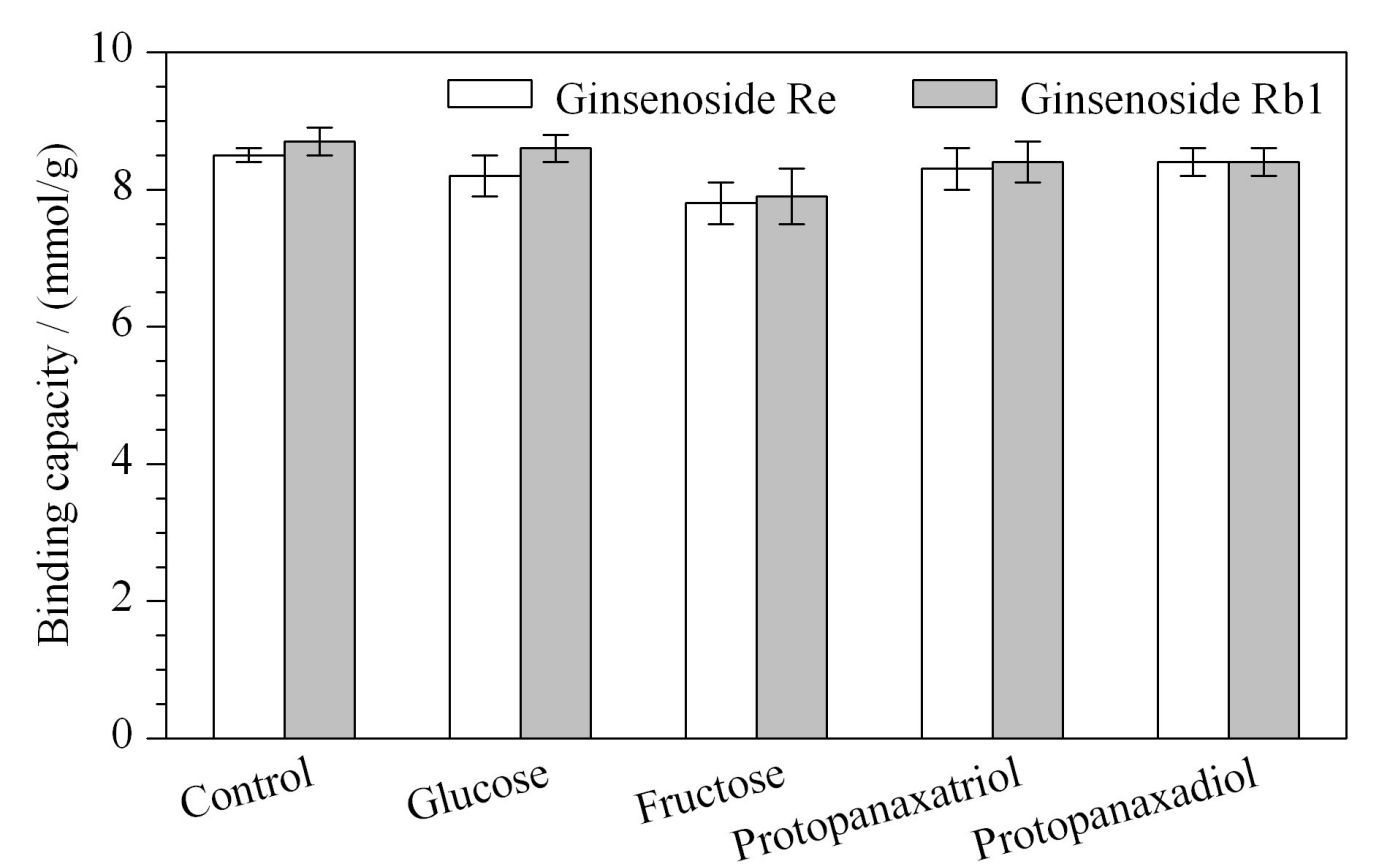
竞争物质对已结合人参皂苷Re和Rb1的PEI-BA-MNPs结合容量的影响(*n*=3)

### 2.5 重复使用性

如图6所示,以人参皂苷Re为例,PEI-BA-MNPs重复使用10次后,吸附容量虽然有所下降,但重复5次后仍保持在初始吸附容量的72.8%。造成此现象的原因可能是:在材料制备过程中,硼酸的修饰是依靠席夫碱反应进行的,随后C=N被还原为C-N以增加稳定性。所得材料可能是存在未被还原的C=N,造成重复使用过程中硼酸被洗脱下来。此结果表明,PEI-BA-MNPs能在一定的使用次数内维持良好的稳定性和重复使用性,后续需要继续优化材料制备过程以进一步提高材料的稳定性。

**图 6:**
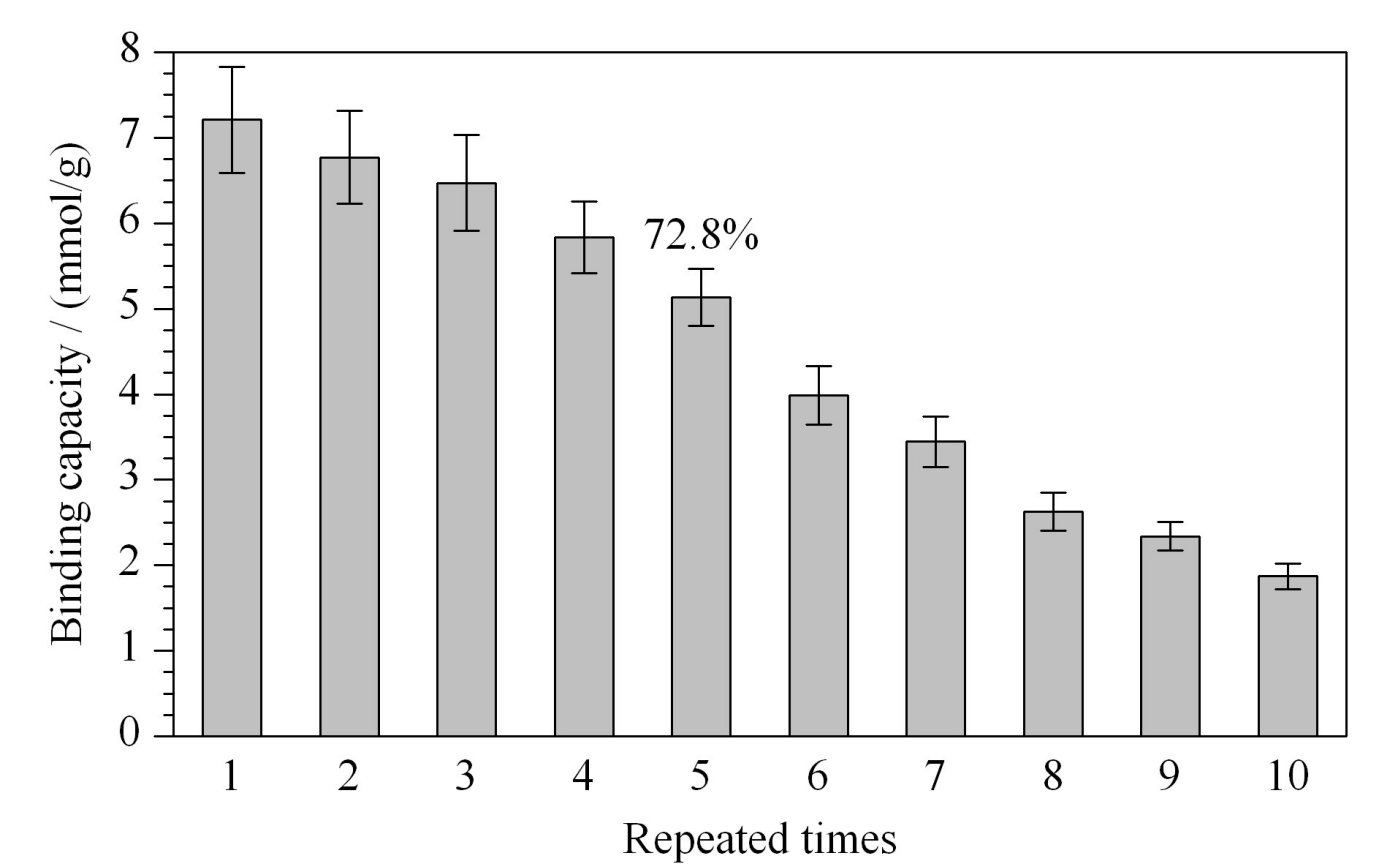
PEI-BA-MNPs重复使用次数与其结合容量的关系(*n*=3)

### 2.6 方法验证

在上述优化条件下以人参皂苷Re和Rb1为代表,对该方法进行了评价。在50~800 μg/L范围内人参皂苷Re的峰面积*y*与质量浓度*x*(μg/L)的线性回归方程为*y*=419*x*+7444,相关系数(*R*^2^)为0.9681,线性良好;人参皂苷Rb1的峰面积*y*与质量浓度*x*(μg/L)的线性回归方程为*y*=418*x*+8011,相关系数(*R*^2^)为0.9799,线性良好。以*S/N*为3和10分别确定检出限(LOD)和定量限(LOQ),结果为20 μg/L和50 μg/L。

实验采用在阴性样品中添加3种不同浓度的人参皂苷Re标准溶液,每个水平平行测定6次,测得方法的回收率和相对标准偏差(RSD),用以评价方法的准确性。所得结果见表1。结果显示,3种水平下的加标回收率为91.5%~117.3%,RSD为7.2%~13.4%。

**表 1:** 样品中人参皂苷Re的加标回收率及精密度(*n*=6)

Spiked level/ (mg/L)	Ginsenoside Re	
	Recovery/%	RSD/%
0.1	117.3	13.4
1	91.5	7.2
10	93.7	10.9

此外,还以不同浓度的人参皂苷Re(10 μg/L、50 μg/L、500 μg/L、1 mg/L、10 mg/L、50 mg/L)为代表,对PEI-BA-MNPs磁性固相萃取和直接进标准样两种方式的信噪比进行了考察,如图7所示,在直接进样中,在1 mg/L时信噪比为7,接近检出限。而经过PEI-BA-MNPs磁性固相萃取后,使用同样的仪器可以检测到质量浓度为20 μg/L的样品。与直接进样的分析方法相比,本方法的灵敏度提高了约50倍,说明PEI-BA-MNPs对人参皂苷Re有良好的富集作用。

**图 7:**
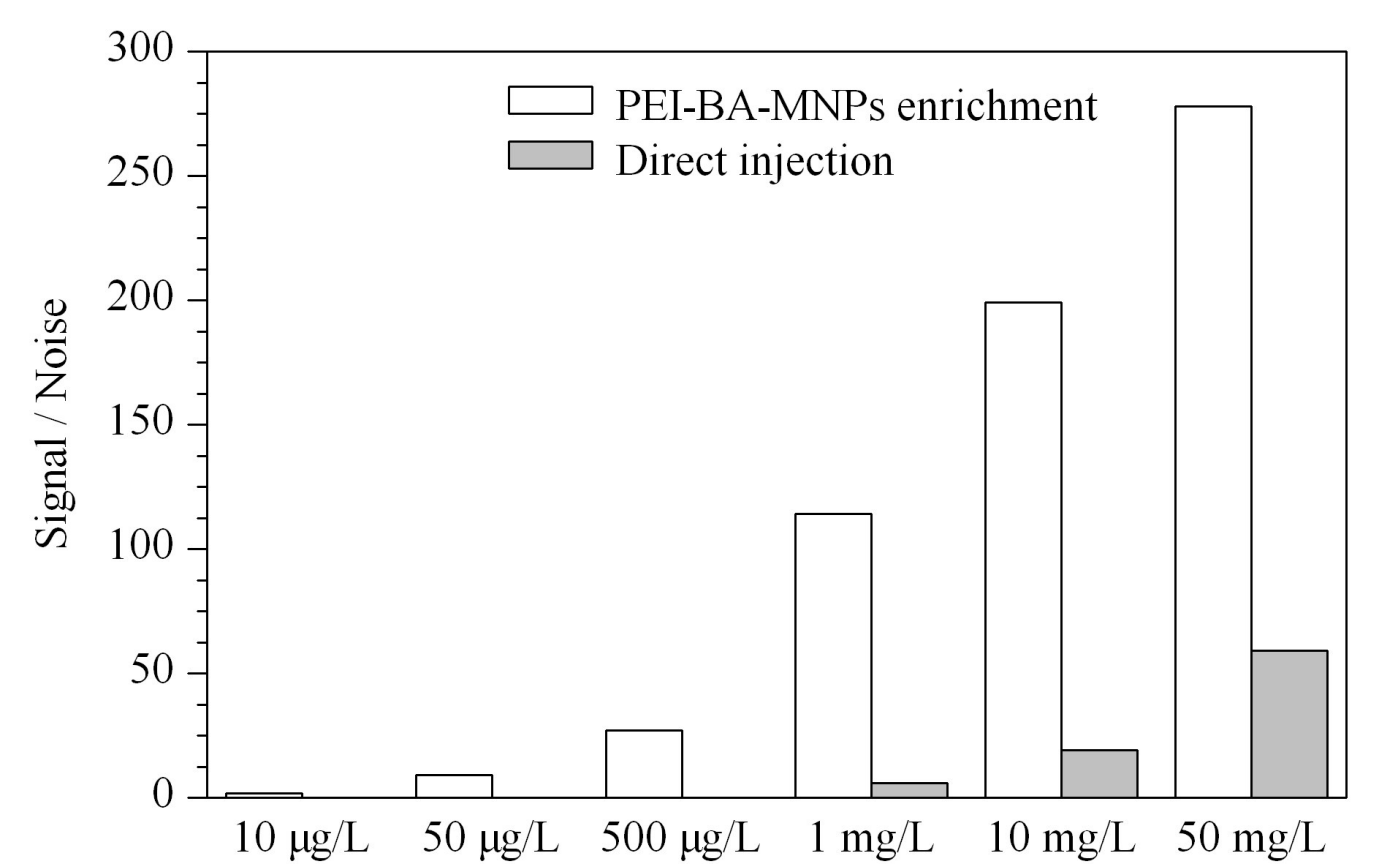
不同浓度人参皂苷Re采用不同预处理方法在仪器中的信噪比对比

### 2.7 实际样品中的应用

启脾口服液中含有人参皂苷成分,本工作选用启脾口服液验证上述方法在实际样品中的应用。启脾口服液分别按照1.3节中所述方法和ChP2015中的标准方法进行处理。如图8所示,富集后样品的色谱图中干扰成分的色谱峰显著减少,可有效地提高样品检测的准确度。同时,本方法中的样品前处理方法相较于ChP2015中有着极大的简化^[28]^。最后,使用ChP2015标准方法进行对比,采用本方法测定的启脾口服液中人参皂苷Re含量为0.27%,而ChP2015标准方法检测出Re的含量为0.31%。结果显示,采用本文中提出的样品前处理技术富集人参皂苷能得到令人满意的效果。

**图 8:**
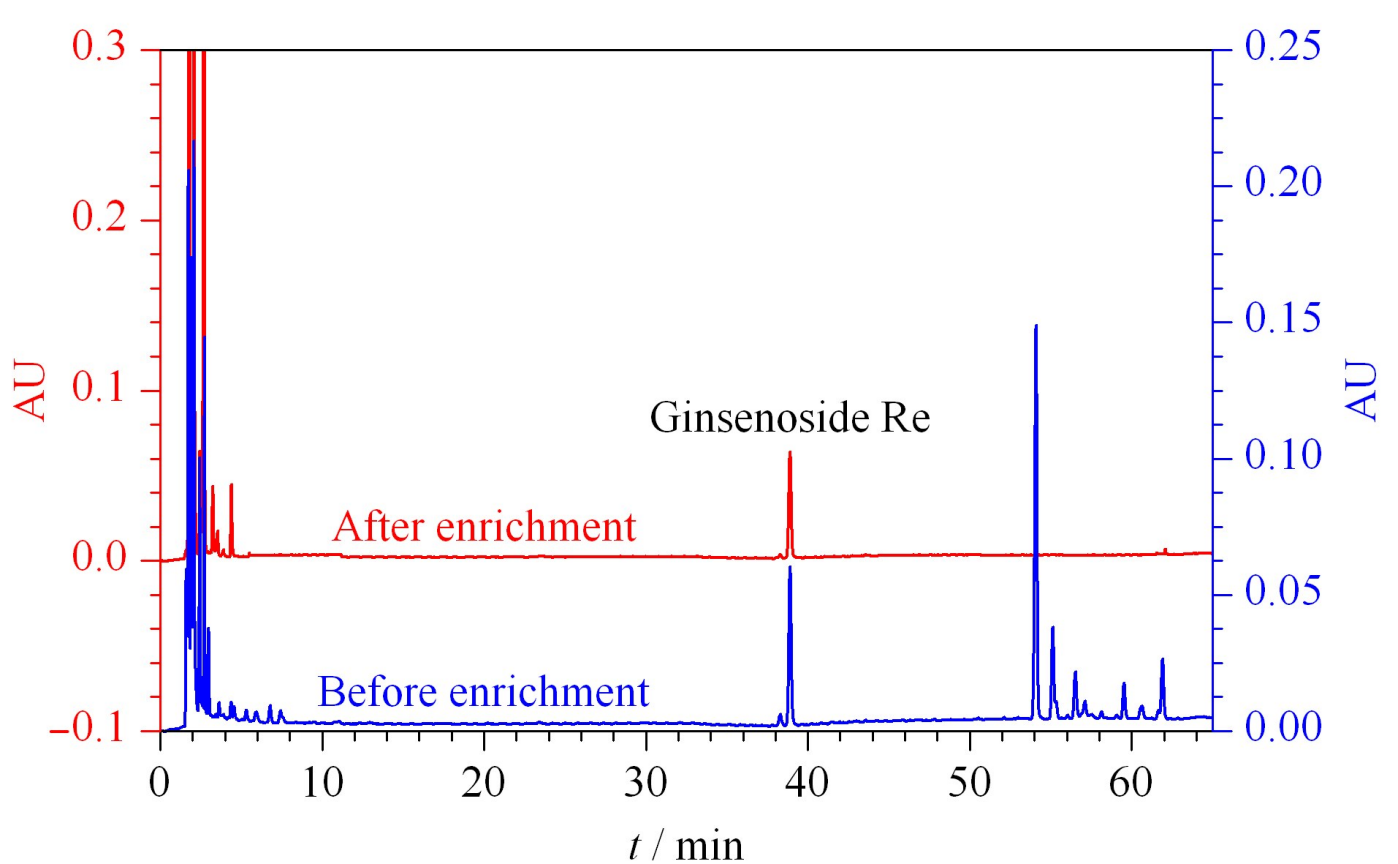
启脾口服液经由PEI-BA-MNPs富集前后的高效液相色谱图

## 3 结论

本研究基于硼亲和作用原理,合成了一种新型磁性纳米颗粒PEI-BA-MNPs,其有均匀的尺寸大小、良好的磁性性能且制备方法简单、分散性良好。将其作为磁性固相萃取吸附剂,结合高效液相色谱分析,建立了一种有效测定实际样品中人参皂苷的方法,可以有效简化药典中复杂的样品预处理步骤。本方法在针对皂苷类的药物分析方面具有较高的应用价值和广阔的应用前景。
